# Letter to the Editor regarding “blood culture bottle shortage mitigation efforts: analysis of impact on ordering and patient impact” by Doern et al.

**DOI:** 10.1017/ash.2025.10053

**Published:** 2025-07-11

**Authors:** Anuschka Y. van der Zaag, Amber G. den Hollander, Sheena C. Bhagirath, Prabath W.B. Nanayakkara

**Affiliations:** Department of Internal Medicine, Section General Internal Medicine, Amsterdam UMC, VU University, Amsterdam, Netherlands

Dear Editor,

We read with great interest the article entitled “Blood culture bottle shortage mitigation efforts: analysis of impact on ordering and patient impact” by Doern et al.^1^ We fully share the authors` view that excessive blood culture collection can be reduced through decision support without negatively impacting patient care. We also commend the authors’ for their swift and effective strategy to reduce the number of blood culture analyses in times of blood culture bottle shortage.

However, we find the conclusion that the measures implemented by Doern et al have not adversely affected patient care to be premature. Firstly, no patient outcomes were measured beyond blood culture positivity. While an increasing positivity rate may indicate a reduction in unnecessary or negative cultures, it remains unclear whether this increase is proportionate or appropriate. Crucial outcomes such as repeat emergency department visits, delayed or ineffective antibiotic treatment, and mortality were not assessed. Assessing these outcomes is essential to ensure patient safety.^2^ Second, the number of single-set blood culture orders did increase during the period of resource constraints. As the authors note, this was an unintended effect of the interventions. Although they plan to address this through ongoing education, it remains unclear whether this shift in ordering practice may have had a negative impact on patient care.

We do acknowledge that evaluating such outcomes within a limited timeframe is very challenging and recognize that prompt action was necessary. However, we recommend that these outcomes be considered in future research. To ensure that patient safety is not compromised by decision support tools it is essential to include outcome measures relevant to its clinical context.^2–4^ Future research should therefore include these outcome measures, ideally within a randomized controlled setting to minimize potential bias. Our research team has developed and extensively validated a machine learning based prediction model to estimate the likelihood of positive blood cultures in the emergency department.^5–7^ This model is currently being evaluated in a non-inferiority randomized controlled trial to ensure effectiveness and safety.^8^ We strongly believe that studies of this kind are absolutely necessary before implementing similar decision support tools in patient care.

Diagnostic stewardship, especially in the context of material shortage, is an important research area. However, more extensive evaluation of decision support tools is imperative before conclusions about their effect on patient safety.
